# Can cerebellar and brainstem apparent diffusion coefficient (ADC) values predict neuromotor outcome in term neonates with hypoxic-ischemic encephalopathy (HIE) treated with hypothermia?

**DOI:** 10.1371/journal.pone.0178510

**Published:** 2017-07-07

**Authors:** Gemma Arca-Díaz, Thomas J. Re, Marie Drottar, Carmen Rosa Fortuno, Katyucia De Macedo-Rodrigues, Kiho Im, Josep Figueras-Aloy, Patricia Ellen Grant

**Affiliations:** 1 Service of Neonatology, Agrupació Sanitaria Hospital Clinic- Hospital Sant Joan de Déu, University of Barcelona, IDIBAPS, Barcelona, Spain; 2 Fetal-Neonatal Neuroimaging and Developmental Science Center, Children's Hospital Boston, Boston, Massachusetts, United States of America; 3 Department of Neurology, Fetal-Neonatal Neurology Program, Boston Children’s Hospital, Boston, Massachusetts, United States of America; Henry Ford Health System, UNITED STATES

## Abstract

**Background and purpose:**

To determine the apparent diffusion coefficient (ADC) in specific infratentorial brain structures during the first week of life and its relation with neuromotor outcome for Hypoxic-ischemic encephalopathy (HIE) in term neonates with and without whole-body hypothermia (TH).

**Materials and methods:**

We retrospectively evaluated 45 MRI studies performed in the first week of life of term neonates born between 2010 and 2013 at Boston Children's Hospital. Selected cases were classified into three groups: 1) HIE neonates who underwent TH, 2) HIE normothermics (TN), and 3) controls. The neuromotor outcome was categorized as normal, abnormal and death. The ADC_mean_ was calculated for six infratentorial brain regions.

**Results:**

A total of 45 infants were included: 28 HIE TH treated, 8 HIE TN, and 9 controls. The mean gestational age was 39 weeks; 57.8% were male; 11.1% were non-survivors. The median age at MRI was 3 days (interquartile range, 1–4 days). A statistically significant relationship was shown between motor outcome or death and the ADC_mean_ in the vermis (*P* = 0.002), cerebellar left hemisphere (*P* = 0.002), midbrain (*P* = 0.009), pons (*P* = 0.014) and medulla (*P* = 0.005). In patients treated with TH, the ADC _mean_ remained significantly lower than that in the controls only in the hemispheres (*P* = 0.01). In comparison with abnormal motor outcome, ADC_mean_ was lowest in the left hemisphere (*P* = 0.003), vermis (*P* = 0.003), pons (*P* = 0.0036) and medulla (*P* = 0.008) in case of death.

**Conclusion:**

ADC_mean_ values during the first week of life in the left hemisphere, vermis, pons and medulla are related to motor outcome or death in infants with HIE either with or without hypothermic therapy. Therefore, this objective tool can be assessed prospectively to determine if it can be used to establish prognosis in the first week of life, particularly in severe cases of HIE.

## Introduction

Neonatal hypoxic-ischemic encephalopathy (HIE) is a major worldwide problem affecting 0.5-1/1000 live births in developed countries. Mortality rates range from 10 to 60%, and long-term neurodevelopmental sequelae are present in 23% of survivors. [1–3] Recent data show that therapeutic hypothermia (TH) may improve the outcome of neonates with HIE and is becoming routine. [4–9]

Diagnostic imaging includes the use of conventional and diffusion-based MRI, with the later capable of identifying ischemic HIE lesions in the acute phase, several days earlier than the former. [10,11] Diffusion MRI, including diffusion-weighted imaging (DWI) and quantitative apparent diffusion coefficient (ADC) mapping, may also provide additional early diagnostic and prognostic information; several studies report an association between reduced ADC values in the posterior limb of the internal capsule (PLIC) as well as an association between reduced ADC values in the basal ganglia and thalami (BGT) and neurological sequelae. [12–21]

Qualitative assessment of brainstem injury on T1 and T2 images in neonates with HIE may provide information on injury severity and risk of death, but objective quantitative data such as ADC values are lacking. [22] These infratentorial areas have high concentrations of excitatory neurotransmitters (e.g., glutamate) and are especially vulnerable to the profound hypoxia-ischemia that is typical in HIE. The cerebellum acts as a satellite system of established cortico-basal ganglia networks in neonates. It is involved in motor and cognitive functions, and newborns with BGT lesions can show reduced cerebellar growth during the first year. [23–27] Recently, Pelzer EA et al [25] confirmed that dentato-thalamo-striato-pallidal and subthalamo-cerebellar connections exist in the human brain, and a study by Connolly and co-authors[26] showed that the high T2 signal intensity in the anterior lobe of the vermis probably represents gliosis secondary to hypoxia/ischemia and is related to the severity of damage in term infants.

In light of these findings, the aim of this study was to determine whether apparent diffusion coefficient (ADC) measurements in specific infratentorial structures during the first week of life can predict neuromotor outcome in both neonates treated with whole-body therapeutic hypothermia (TH) and those without whole-body therapeutic hypothermia or normothermics (TN).

## Materials and methods

An electronic search was performed of the neonatal and neurology databases at Boston Children's Hospital (BCH) for neonatal MRI studies performed over the 3-year period from March 2010 to June 2013. BCH’s NICU is a 24-bed tertiary referral center for critically ill newborns, premature newborns and newborns who require complex medical and surgical care. The Hospital Ethics Committee of BCH approved this study, which was a retrospective review and analysis of clinically indicated MRI studies and the corresponding clinical charts.

### Patients

Forty-five clinical charts of patients born at term (GA ≥ 36 weeks) with MRIs performed for suspected HIE or clinically suspected neonatal seizures within the first week of life were identified. These patients were categorized as: 28 HIE who underwent TH, 8 HIE normothermics (TN), and 9 controls (admitted to NICU for suspected clinical seizures and no clinical signs of symptoms of HIE).

The exclusion criteria included evidence of metabolic disease, congenital infection, major malformations, alcohol or drug embryopathies, hydrops, chromosome abnormalities, and MRI evidence of long-standing brain damage or developmental abnormality.

Newborns were considered for active whole-body therapeutic hypothermia (TH) if they met a combination of diagnostic criteria including clinical, laboratory and amplitude-integrated electroencephalogram (aEEG) findings, as established by the first two large published trials of therapeutic hypothermia for neonatal HIE. [4,5] The criteria for treatment within 6 hours after birth included (1) gestational age ≥36 weeks and birth weight ≥ 2000g; (2) evidence of fetal distress including history of acute perinatal event; (3) evidence of neonatal distress as shown by at least one of the following: Apgar score ≦ 5 at 10 minutes, pH ≦ 7.0 within 1 hour of birth or base deficit < 16mEq/L, or need for ventilation for at least 10 minutes after birth; and (4) evidence of neonatal encephalopathy diagnosed by both neurological examination and aEEG on admission to BCH. The normothermic babies did not meet the inclusion criteria at the time of initial clinical and electroencephalographic evaluation. TH was targeted at 33.5° for 72 hours, followed by 12 hours rewarming at 0.25°C/hour.

### Neuromotor outcome

All surviving infants underwent developmental and neurologic examinations performed by experienced and specially trained neonatologists, pediatricians, or child neurologists. Neonatal clinical classification of HIE was retrospectively graded according to Sarnat and Sarnat [27](*stage 1*: mild encephalopathy lasting 24 hours with normal EEG; *stage 2*: moderate encephalopathy, lethargy, hypotonia and seizures; *stage 3*: severe encephalopathy, coma and if hypotonic with decreased or absent reflex activity and severely depressed EEG findings). In 75% of our cohort of surviving children, there was suspicion of cerebral palsy (CP), and an assessment of neuromotor skills level was performed according to the GMFCS (Gross Motor Function Classification System) developed by Palisano and colleagues. [28] Patients with normal neurodevelopment, normal neurological examination findings and normal cognitive developmental history as well as children without cerebral palsy were not included on this scale. Seven infants younger than six months at the time of follow-up were not classified in this system. GMFCS levels I-III (mild and moderate CP) correspond to patients 6 to 24 months old who walk as their preferred method of mobility, whereas children at levels IV and V (severe CP) usually require a wheelchair for mobility. In our study, the outcome classification took into account all available information from all follow-up visits. We considered three possible neuromotor outcomes: normal (without CP), abnormal (with CP) and death.

### Imaging

During this study period, routine clinical neonatal MRI studies were performed on a 3.0T Siemens Trio (Siemens, Erlangen, Germany) scanner with a 32-channel commercial head coil (Siemens, Erlangen, Germany). The routine neonatal protocol included conventional sequences: Axial TURBO T1 (SE 2500/9); Fast Spin Echo T2 weighted (FSE8000/90) sequences (both with a 128 x128 matrix, 2.5 to 7-mm slice thickness, and 2 averages). The routine clinical DTI was a single-shot multi-level echo planar sequence with 40 slices (repetition time (TR) 8000 msec, echo time (TE) 88 msec, inversion Time (IT): -1 msec, field of view (FOV) 25 x 30cm, 30 gradient directions at b values of 1000 mm^2^/s, 5 gradient directions at b = 0 mm^2^/s, voxel sizes: 2.00, 2.00, 2.00). The 45 included infants received a first MRI within one week of delivery, and these MRIs were analyzed. Twenty infants were rescanned during the first month for confirmation/follow-up. For those on TH, induced hypothermia was continued during MRI. All neonates were scanned during natural post-feeding sleep while blanket-wrapped and earmuff-fitted. MRI was performed according to BCH safety and quality guidelines. Body temperatures were recorded before and after MRI scans in infants undergoing TH.

### Image analysis

**Fig 1:**
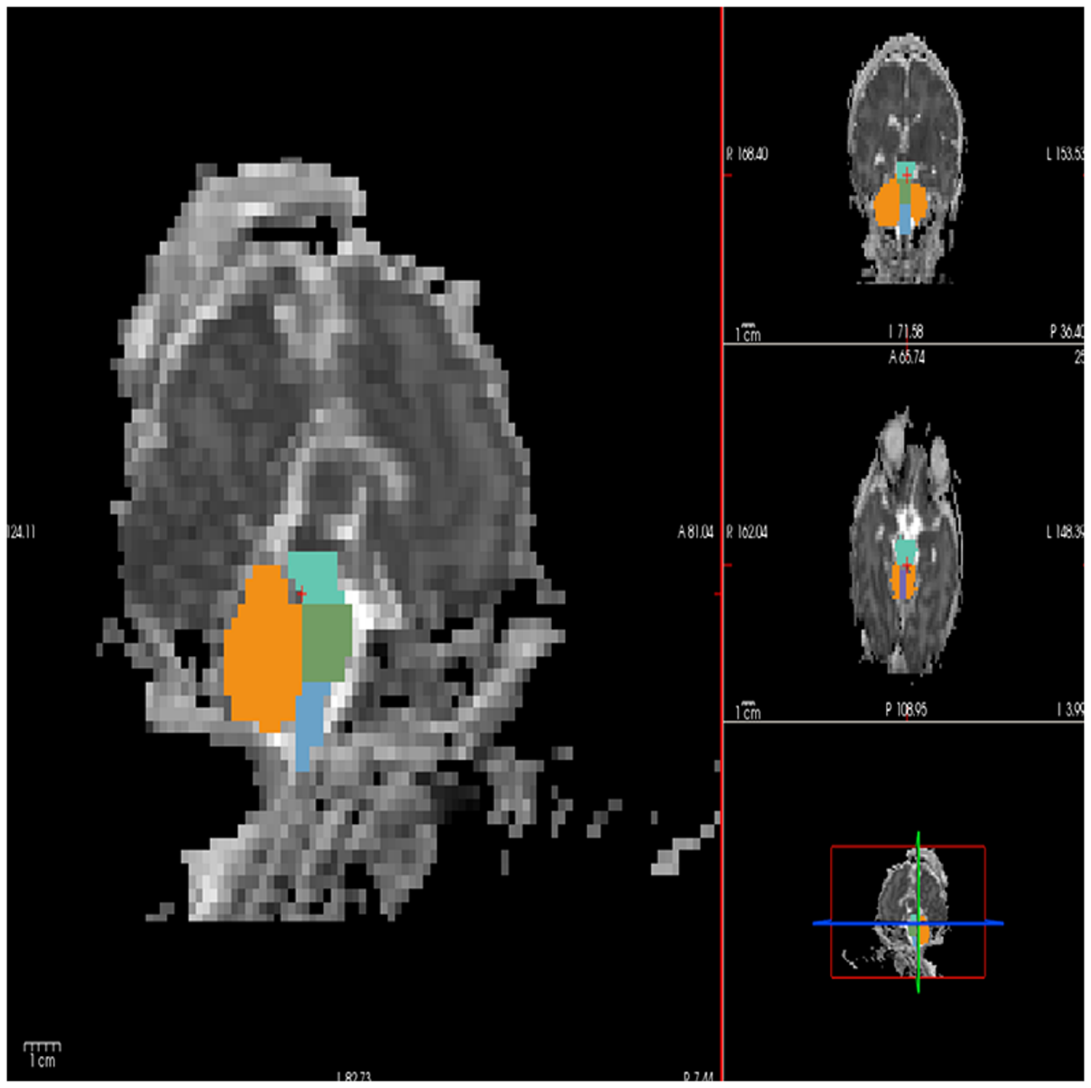
ROI locations on an ADC map within free view. A semiautomated segmenting tool within the FreeSurfer software package. ROIs were drawn for the hemispheres of the cerebellum (orange), vermis (violet), midbrain (turquoise- blue), pons (green) and medulla (light blue). Left sagittal view, right from top to bottom, coronal, axial and triplane image.

Diffusion tensor images (DTI) were imported from PACS using the ChRis system (Children's Research Interpretation System). For each DTI data set, the FSL/FLIRT co-registration tool (fsl.fmrib.ox.ac.uk) was used to align all 35 gradient directions and the b = 0 images and to remove any eddy current artifacts. DTI data sets were then processed by TrackVis to create ADC and FA maps. Six anatomical infratentorial substructures were chosen for ADC analysis: the cerebellar left hemisphere, cerebellar right hemisphere, vermis, midbrain, pons and medulla. These structures were manually delimited as regions of interest (ROIs) on the ADC map for all 45 subjects by author G.A. (a neonatologist with experience in neonatal neuroimaging) using FreeView. Segmentations were performed in a similar manner as described previously for volumetric T1 segmentations [29] (Fig 1) and were reviewed by author M.D. (Project Coordinator with 10 years of neuroimaging experience). The maximum intra-observer and inter-observer (G.A. and M.D.) errors for ADC measurements were estimated to be 3–5%. The MRIs were reviewed, without clinical history knowledge, by a highly experienced neuroradiologist (P.E.G.). The ADC_mean_ values were calculated for each of the ROIs.

### Statistical analysis

Quantitative ADC data were analyzed using MATLAB R2013b (Version 8.3; http://www.mathworks.it). Intra-observer error was measured using Kappa statistics. Comparisons between groups were performed using a Kruskal Wallis test for non-normally distributed data. Categorical data were analyzed with a chi-square test or Fisher’s exact test as appropriate. Continuous variables in normally distributed data were analyzed using the Oneway test with the Scheffé test for multiple comparisons. The relationships between ADC values in each ROI with motor outcome and death were assessed by the Oneway test. Differences with *P* level< 0.05 were considered significant. You can find the database in S1 File.

## Results

### Patients

Forty-five infants were included in the retrospective study: 28 moderate-severe HIE TH, 4 moderate-severe HIE TN, 4 mild HIE and 9 controls (mean estimated GA: 39.0 weeks, 26 males). The first MRI was performed at a median of 3 days post-delivery (interquartile range, 1–4 days). The Sarnat staging was as follows: 4 stage 1, 18 stage 2 (15 HT, 3 TN) and 14 stage 3 (13 TH, 1TN). Information regarding the demographic characteristics of the study group is shown in Table 1. The median age at follow-up was 18 (range 6–24) months. There were 5 non-survivors (11.1%), all of who died on the first day of life. Of the surviving infants, 20 had CP, including 13 mild CP (Level I), 6 moderate CP (Level II/III), and 1 severe CP.

**Table 1:** General characteristics of the study population.

	Control (n = 9)	HIE (n = 36)			P- value*
		Mild (n = 4)	Moderate (n = 18)	Severe (n = 14)	
**Birth weight (g)**	3428 ± 381	3445 ±371	3250 ±460	3737±590	0,062
**Gestational age (w)**	39,67 ±1,22	38,50 ±0,57	38,39 ±1,37	39,57±1,34	0,032
**HC (cm)**	34,44 ±0,63	36,12±2,35	34,44±1,24	35,57±1,55	0,036
**L (cm)**	52,33 ±2,10	47,50±4,43	50,29±2,78	51,00±2,71	0,044
**Cord pH**	7,31±0,127	7,17±0,00	6,96±0,166	6,85±0,144	0,004
					P- value**
**Gender (male)**	7(77,8%)	3 (75,0%)	10 (55,6%)	6 (42,9%)	0,078
**Death**	0	0	0	5(35,7%)	0,011
**Apgar (min)**					P- value***
**1**	8 (7–9)	3 (2–3,5)	1(1–4)	1(0–1)	<0,001
**5**	9 (9–9)	8 (7–8,5)	3(2–5)	3(1–3)	<0,001
**10**	10 (9–10)	10 (8,5–10)	5(4–7)	4(3–5)	<0,001
**Lactate (mmol/L)**	NA	NA	4,95(3,8–5,85)	12,1(3,6–18)	0,165
**Days of life at MRI [Range]**	4(2–4)	3(2–3)	4(1–4)	2(1–4)	0,663
	[1–6]	[1–3]	[1–6]	[1–5]	

Mean±SD; Percentile 50 (Percentile 25-Percentile 75), N (%), [range].

*Oneway and Scheffé between each pair or

** Chi-square or

*** Kruskal—Wallis. HIE, Hypoxic-ischemic encephalopathy; HC, head circumference; L, length; w, weeks; cm, centimeters; NA, Not available.

### ADC measurements

Table 2 shows the ADC_mean_ values and standard deviations (μm^2^/ms) for each infratentorial ROI for all patients. No significant differences in ADC_mean_ were observed. As shown in Table 3, the ADC_mean_ values in the cerebellar hemispheres were lower when hypothermia treatment was administered. The ADC_mean_ values based on outcome are shown in Table 4. There was a significant reduction of ADC_mean_ values according to neuromotor outcome (normal, abnormal and non-survivor) in the vermis (*P* = 0.002, left hemisphere cerebellum (*P* = 0.002), midbrain (*P* = 0.009), pons (*P* = 0.014) and medulla (*P* = 0.005). Significantly lower ADC_mean_ values were observed in all ROIs except the right hemisphere in non-surviving patients compared with survivors.

**Table 2:** The ADC_mean_ (SD) x10^-3^/mm^2^/second at first MRI in six regions of interest (ROIs) in all infants.

Brain area (ROI)	Control (n = 9)	HIE (n = 36)			*P value
		Mild (n = 4)	Moderate (n = 18)	Severe (n = 14)	
**RH**	1,19±0,100	1,31±0,136	1,20±0,086	1,16±0,123	0,144
**LH**	1,20±0,094	1,34±0,110	1,22±0,104	1,17±0,131	0,069
**Vermis**	1,15±0,095	1,20±0,148	1,16±0,095	1,13±0,134	0,712
**Midbrain**	1,16±0,071	1,14±0,128	1,19±0,095	1,13±0,125	0,4
**Pons**	1,18±0,073	1,21±0,109	1,18±0,092	1,18±0,108	0,958
**Medulla**	1,32±0,142	1,43±0,103	1,37±0,100	1,35±0,146	0,535

Mean+/-SD

*Oneway test.

HIE, hypoxia-ischemia encephalopathy; ROI, Region of Interest; RH, Right cerebellar hemisphere; LH, Left cerebellar hemisphere.

**Table 3:** The ADC_mean_ (SD) x10^-3^/mm^2^/second at first MRI in six regions of interest (ROIs) in newborn with moderate and severe HIE with and without hypothermia.

	HIE moderate—severe (n = 32)		
Brain area (ROI)	HT (n = 28)	No HT (n = 4)	P- value*
**RH**	1,17±0,092	1,30±0,120	0,016
**LH**	1,18±0,108	1,33±0,095	0,014
**Vermis**	1,14±0,109	1,16±0,152	0,775
**Midbrain**	1,15±0,109	1,25±0,101	0,09
**Pons**	1,17±0,095	1,24±0,107	0,192
**Medulla**	1,36±0,124	1,41±0,084	0,424

Mean +/- SD

*Oneway test. HIE, hypoxia-ischemia encephalopathy; ROI, Region of Interest; RH, Right cerebellar hemisphere;

LH, Left cerebellar hemisphere. HT, hypothermia

**Table 4:** The ADC_mean_ (SD) x10^-3^/mm^2^/second at first MRI in six ROIs and neuromotor outcome.

	Neuromotor outcome			
Brain area (ROI)	Normal (n = 13)	Abnormal (n = 20)	Died (n = 5)	P- value*
**RH**	1,21 ±0,100	1,23 ±0,097	1,10 ±0,113	0,05
**LH**	1,24 ±0,104	1,25 ±0,096	1,06 ±0,106	0,002
				N-D: 0,008
				A-D: 0,003
**Vermis**	1,18 ±0,104	1,17 ±0,095	1,00 ±0,064	0,002
				N-D: 0,004
				A-D: 0,003
**Midbrain**	1,21 ±0,100	1,16 ±0,091	1,05 ±0,092	0,009
				N-D: 0, 009
**Pons**	1,22 ±0,084	1,20 ±0,077	1,09 ±0,081	0,014
				N-D: 0,016
				A-D: 0,036
**Medulla**	1,40 ±0,110	1,40 ±0,072	1,24 ±0,125	0,005
				N-D: 0,010
				A-D: 0,008

*Oneway-Scheffé. NS: not significant. RH: Right cerebellar hemispheres. LH: Left cerebellar hemisphere. N: Normal, A: Abnormal, D: Died

## Discussion

Previous studies have examined the possible diagnostic and prognostic value of early diffusion MR-derived ADC for suspected neonatal HIE but have yielded inconsistent results. [30–33] The current work offers new perspectives regarding ADC and HIE in neonates. The present study used the latest clinical MR technology utilizing a 3.0T scanner and higher b value (1000 s/mm^2^) to maximize the accuracy of the ADC measurements. Moreover, it focused on several distinct substructures of the brain rather than measuring the mean ADC across large brain regions. Our results indicated variability in ADC across structures, and thus it is understandable that ADC averaging over large brain regions might produce unreliable results. As the routine use of hypothermia therapy is relatively recent, this work is the first to consider the association of such therapy with ADC measurements. Finally, while previous ADC studies have demonstrated the prognostic value of ADC measured in supratentorial structures, specifically the posterior limb of the internal capsule (PLIC), basal ganglia and thalamus, [17,34,35] this study focuses on subtentorial structures.

Conventional T1- and T2-weighted magnetic resonance imaging (MRI) assists the definition of the nature and extent of perinatal brain injury but must be interpreted in the context of its timing. Conventional MRI is an excellent predictor of outcome if performed after one week following the hypoxic-ischemic event. [10,11]. Diffusion MRI, including diffusion-weighted imaging (DWI) and quantitative apparent diffusion coefficient (ADC) mapping, is more useful than conventional MRI in the early detection of perinatal brain hypoxia-ischemia. Diffusion MRI permits the identification of intracellular cytotoxic edema present in the acute phase of HIE lesions two to five days before such lesions are apparent in conventional MRI. In addition, diffusion MRI may provide additional diagnostic and prognostic information during the acute phase, when important clinical decisions must be made.

We found eight studies and one meta-analysis that calculated ADC values in the posterior fossa. [12,13,19,20,21,30,35–37] Our results can only be compared with a few of these studies because, in contrast to our study, these studies did not distinguish between the different infratentorial structures and did not classify subjects according to the severity of HIE. Sener et al, [31] who studied midbrain ADC_mean_ values across many ages, reported a mean ADC of 1.0±0.10 μm^2^/ms for infants 0 to 2 years of age. Liauw et al [19] measured an ADC of 1.075 [range, 0.92–1.24] μm^2^/ms in the midbrain in control infants. We report a slightly higher ADC_mean_ of 1.16 μm^2^/ms [range, 1.03–1.29] for the subtentorial region; however, this value could be considered consistent with Sener's finding based on the younger age of our subjects (all neonates). Our value coincides with the ADC_mean_ value measured by Vermeulen et al [20] for a group of 4 healthy infants (1.16 μm^2^/ms).

Rutherford M et al [13] compared the ADC values in different brain regions between 15 control and 63 symptomatic term-born neonates affected by HIE. In contrast to our study, the HIE neonates were not classified by severity, and all patients were normothermics. In the control group, the ADC_median_ in the vermis was 0.97 μm^2^/ms [range, 0.8–1.2], and the overall subject value was 0.98 μm^2^/ms [range, 0.7–1.2], with no significant differences between the groups. Ward et al [36] measured ADC values in 13 different points of the cerebellar hemispheres and vermis in 20 HIE normo-thermic neonates as 1.10 μm^2^/ms [range, 0.97–1.18] and in controls as 0.90 μm^2^/ms [range, 0.80–1.14]. These results are lower than our findings for the vermis in controls (1.16 μm^2^/ms [range: 1.03–1.33]), but this difference might be attributable to the use of higher b value in the present study.

Martínez-Biarge et al [22] reported that in newborns with HIE, the best predictor of subsequent death was the presence of apparent brainstem injury. In our study, the non-survivors exhibited a lower ADC_mean_ in all ROIs compared with survivors. Interestingly, the vermis exhibited the highest ADC_mean_ difference between non-survivors and survivors. We have previously shown that cerebellar growth is reduced after perinatally acquired BGT lesions and that this reduction is most marked within the vermis. [24] It is likely that reduced cerebellar growth occurs as a secondary phenomenon in infants with perinatally acquired BGT lesions. The cerebellar cortical (Purkinje) cells in the cerebellar vermis are particularly prone to ischemic damage due to their inability to generate energy during anoxia, a factor that increases the damage produced by initial hypoxia. [38] Impaired growth of vermis is likely a secondary effect related to damage in other remote but connected areas of the brain or so-called “diaschiasis”. Accordingly, Le Strange et al [24] observed an apparently normal cerebellar hemisphere growth pattern in HIE patients but a tendency toward vermian atrophy secondary to thalamic edema. In our study, the ADC_mean_ values of the six ROIs were not useful for differentiating normal outcomes from abnormal motor outcomes or death because the reduction in the structures was only significant in the most severe cases, who died. These findings should be considered with caution given our small sample size, and further studies are needed before reaching a final conclusion.

No other study has classified ADC values according to the severity of HIE, and none of the reviewed studies refer to hypothermic therapy, which was not commonly used at the time of their publication. Only Massaro et al [39] calculated ADC values in HIE neonates treated with hypothermia but only calculated ADC for the BGT. Our study shows that all ADC_mean_ values in each infratentorial ROI in the comparison of the two treatment conditions (hypothermia *versus* normothermia) were lower in HT infants, but the differences were only statistically significant in the cerebellar hemispheres. The mean diffusivity likely has a weak dependence on temperature (2.4% per 1°C change), and diffusion measurements obtained during the cooling period are expected to be lower than those obtained at normal body temperature. [40] In our study, we registered the temperatures before and after the MRI process but not during. Further studies are needed to assess the effect of hypothermia on ADC, particularly in non-perfused tissues such as HIE lesions.

Diffusion weighted images obtained in the time frame between the 2nd and 4th days post-partum have been shown to more reliably reflect the extent of injury than those performed outside this time frame. Our study focused on infants who received their initial MRI at 3 days post-partum, within this ideal time frame. In agreement with the literature, we observed a significant tendency for ADC_mean_ to increase over time between the initial and follow-up MRI. By day 7, diffusion MR is less sensitive for the detection of perinatal brain injury compared with conventional MR because of transient pseudonormalization or “fogging”, i.e., the diffusion measurements (including ADC) in the ischemic tissue appear to normalize. [39, 40] More recently, Bednarek et al [41] showed that in infants who underwent therapeutic cooling following HIE, the largest reduction in ADC was observed in severely injured infants scanned between the 11th and 12th days post-partum. In our study, no MRIs were analyzed beyond the 7th day post-partum. More studies are needed to better understand the pseudonormalization phenomena in TH infants and aid the interpretation and ideal timing of diffusion-based clinical scans.

## Limitations

The limitations of the present retrospective study include the limited sample size, lack of true controls (our controls were admitted to the NICU for suspected clinical seizures), and inherently subjective and time-consuming method of manually drawing ROIs. It might have been interesting to also study the dentate nuclei, but this structure could not be reliably identified in our diffusion data and was not included. Normal values of ADC may depend on several variables, including field strength, type of head coil, *b*-values used, and temperature of the patient when scanned during hypothermia Although this study considered patient temperatures pre- and post-MRI exam, patient temperature during the exam was not available for all infants. In the present study, in infants examined at the same field strength 3.0 T and with the same DWI *b*-values of 1000 mm^2^/s was used and however, this did not appear to have affected our results. ADC values tend to decrease with an increasing number of directions but we used the same number in this study. Finally, in hypotonic moderate-severe HIE newborns, there is currently no practical clinical test to distinguish hypotonia due to cerebellar lesions (cerebellar hypotonia) from that due to cortical-spinal lesions (cortical-spinal hypotonia), which may have altered the accuracy of our clinical classification.

## Conclusions

Diffusion-based ADC assessment, when combined with conventional MRI data and current clinical tests, including amplitude integrated electroencephalography (aEEG) and somatosensory evoked potentials (SEP), can provide useful early information for neurological outcome. Although further studies are necessary to confirm these findings, early ADC measurements in specific infratentorial structures (particularly the vermis, left cerebellar hemisphere, midbrain and pons) can be indicators of neuromotor outcome, particularly survival, in neonates affected by hypoxic ischemia encephalopathy.

## Supporting information

S1 File Database. This is the database of all patients included in the study.
(SAV)

## Abbreviations

ADC: Apparent diffusion coefficient
aEEG: amplitude-integrated electroencephalogram
BGT: Basal ganglia and thalami
CI: Confidence Interval
CP: Cerebral palsy
DWI: Diffusion-weighted imaging
EEG: electroencephalogram
FOV: Field of view
FSE: Fast spin echo
GMFCS: Gross Motor Function Classification System
HIE: Hypoxic-ischemic encephalopathy
MRI: Magnetic resonance imaging
PLIC: posterior limb of the internal capsule
ROI: Region of interest
SE: Spin Echo Imaging
TCD: Transcerebellar Diameter
TE: Echo time
TH: Hypothermia
TN: Normothermia
TR: Repetition time
VH: Vermis Height
WHS: Watershed areas
WM: White Matter
